# Quality of life of locally advanced pancreatic cancer patients after FOLFIRINOX treatment

**DOI:** 10.1007/s00520-021-06648-1

**Published:** 2021-11-11

**Authors:** Fleur van der Sijde, Laura Schafthuizen, Freek R. van ’t Land, Miranda Moskie, Hanneke W. M. van Laarhoven, Monique van Dijk, Casper H. J. van Eijck

**Affiliations:** 1 Department of Surgery, Erasmus MC, University Medical Center Rotterdam, P.O. Box 2040, 3000 CA, Rotterdam, The Netherlands; 2 Department of Internal Medicine, Erasmus MC, University Medical Center Rotterdam, Rotterdam, The Netherlands; 3 Department of Medical Oncology, Cancer Center Amsterdam, Amsterdam UMC, University of Amsterdam, Amsterdam, The Netherlands

**Keywords:** Pancreatic cancer, FOLFIRINOX, Quality of life, EORTC QLQ-C30

## Abstract

**Background:**

Quality of life in cancer patients might be affected by chemotherapy-induced toxicity. Especially in patients with pancreatic ductal adenocarcinoma (PDAC), with a short life expectancy, fear of poor quality of life is often a reason for both patients and medical oncologists to refrain from further treatment. In this study, we investigated quality of life (QoL), pain, sleep, and activity levels in locally advanced pancreatic cancer (LAPC) patients after FOLFIRINOX treatment.

**Methods:**

A total of 41 LAPC patients with stable disease or partial response were included after completion of at least four cycles of FOLFIRINOX. QoL was measured with the EORTC QLQ-C30 and NRS pain scores. Patients completed the Richards-Campbell Sleep Questionnaire (RCSQ) for five consecutive nights and wore a GENEActiv tri-axial accelerometer (Actiwatch) for 7 days, registering sleep duration, efficiency, and activity.

**Results:**

Mean EORTC QLQ-C30 score for global health status was 78.3 (± 17.3), higher than reference values for cancer patients (*P* < 0.001) and general population (*P* = 0.045). LAPC patients reported few disease-related symptoms. Two patients (5%) reported pain scores > 3. Mean sleep duration was 8 h/night (± 1.2 h) and sleep efficiency 70% (± 9%) with high patient-reported quality of sleep (mean RCSQ score 72.0 ± 11.4). Mean duration of moderate-vigorous activity was 37 min/week (± 103 min/week).

**Conclusions:**

QoL is very good in most LAPC patients with disease control after FOLFIRINOX, measured with validated questionnaires and Actiwatch registration. The fear of clinical deterioration after FOLFIRINOX is not substantiated by this study and should not be a reason to refrain from treatment.

**Trial registration:**

Dutch trial register NL7578.

**Supplementary Information:**

The online version contains supplementary material available at 10.1007/s00520-021-06648-1.

## Introduction

FOLFIRINOX chemotherapy, a combination of fluorouracil, leucovorin, irinotecan, and oxaliplatin, is standard of care for locally advanced pancreatic cancer (LAPC) patients with a good performance status. The median overall survival (OS) of these patients is 24.2 months with FOLFIRINOX [1]. The survival benefit of FOLFIRINOX is greater compared to other chemotherapy regimens, such as gemcitabine [1, 2]. Unfortunately, the response rate of FOLFIRINOX is rather low; approximately 10–30% of patients show significant shrinkage of the primary tumor [3, 4]. However, in most patients, FOLFIRINOX will stabilize the disease and prevent early metastasis [4]. Nevertheless, medical oncologists are still cautious to administer FOLFIRINOX to pancreatic ductal adenocarcinoma (PDAC) patients because of the high toxicity rate [5, 6]. More than half of patients will experience FOLFIRINOX-related toxicity, including nausea and vomiting, diarrhea, fatigue, neuropathy, mucositis, thrombocytopenia, and neutropenia [1, 4, 6]. These toxicity-related symptoms might impact the quality of life of these patients and even be a reason to choose or switch to another, less toxic chemotherapy regimen or stop treatment completely. Quality of life is especially important for patients with a short life expectancy, such as LAPC patients. Whether or not diminished quality of life is worth the survival benefit is of course a very personal decision that should be made by patients themselves, but it is the treating physician that should properly inform them on the pros and cons of (chemotherapy) treatment. Next to chemotherapy-induced toxicity symptoms, patients can of course also suffer from disease-associated symptoms. Depending on the tumor location, patients often report duodenal obstruction, icterus, and exocrine pancreatic insufficiency [7], which might be diminished by the administration of chemotherapy. In addition, it is known that many PDAC patients show signs of sleep problems, anxiety and depression, and pain [7, 8]. However, if patients still report these symptoms, and what their quality of life is after chemotherapy, has not been studied yet.

For that reason, the aim of this article was to investigate the quality of life of LAPC patients after treatment with FOLFIRINOX, based on validated questionnaires for quality of life and sleep, Actiwatch activity and sleep registration, and patient-reported pain scores. Additionally, quality of life, activity, sleep, and pain were compared between patients with short (< 12 months) and long (> 12 months) survival after completion of FOLFIRINOX treatment.

## Materials and methods

### Patient selection

LAPC patients were selected from a single-center, prospective clinical trial (Dutch trial register NL7578) investigating the safety and efficacy of adding IMM-101 immunotherapy, a suspension of heat-killed whole cell *Mycobacterium obuense*, to the treatment for LAPC patients with FOLFIRINOX followed by stereotactic body radiation therapy (SBRT). Patients were included in the study after completion of at least four cycles of FOLFIRINOX and before the start of SBRT combined with IMM-101 between October 2019 and January 2021. Other inclusion criteria were age 18–75 years, World Health Organization (WHO) performance status < 2, and American Society of Anesthesiologists (ASA) classification < III. Patients were excluded if they showed progressive disease during or immediately after FOLFIRINOX, since they were not eligible for radiation therapy anymore. Patients were also excluded when they had received previous chemotherapy other than FOLFIRINOX and if they were or had been treated with immunotherapeutic drugs or immunosuppressive drugs. Also, patients with immunodeficiency, a history of human immunodeficiency virus (HIV) infection, or active hepatitis B or C were excluded. This trial, and the side study on quality of life, was approved by the medical ethics review board (MEC-2019–0219). All patients provided written informed consent, and the study was conducted in accordance with the Declaration of Helsinki.

### Study procedure

Upon inclusion in the study, after completion of FOLFIRINOX and before start of SBRT with IMM-101, patients started wearing a GENEActiv tri-axial accelerometer (Actiwatch) to register their activity and sleep for 7 days and filled out the Dutch language version of the Richards-Campbell Sleep Questionnaire (RCSQ) for 5 consecutive days. In addition, they filled out the Dutch language version of the EORTC QLQ-C30 quality of life questionnaire. Patients reported pain scores using a numeric rating scale (NRS) of 0–10 at time of inclusion as part of routine care.

### Measurement instruments

The European Organization for Research and Treatment of Cancer Quality of Life Questionnaire-C30 (EORTC QLQ-C30) is a validated 30-item questionnaire of self-reported health-related quality of life of cancer patients containing both single- and multi-item measures, including global health status/overall quality of life, five functional scales (physical, role, cognitive, emotional, and social functioning), three symptom scales (fatigue, pain, and nausea/vomiting), and six single items (constipation, diarrhea, insomnia, dyspnea, appetite loss, and financial difficulties). Higher scores for global health status and functional scales suggest better quality of life and functioning, while higher scores for symptoms represent more symptoms and thus worse quality of life [9]. An overview of items in the EORTC QLQ-C30 is presented in Supplementary Table 1.

The validated RCSQ contains five aspects of sleep: sleep depth, falling asleep (sleep latency), number of awakenings, returning to sleep, and overall quality of sleep. Each item is scored on a VAS of 0–100. Higher scores represent better sleep quality. Scores between 0 and 25 represent very poor sleep, scores of 26–50 poor sleep, scores 51–75 good sleep, and 76–100 very good sleep [10–12]. The items of the RCSQ are shown in Supplementary Table 2.

The GENEActiv tri-axial accelerometer (Activinsights, Kimbolton, UK) is a wrist-worn accelerometer that provides raw movement data, light, temperature, and posture change measurements. It measures bed time, rise time, elapsed sleep time, sleep time, sleep efficiency, activity levels, and the amount of time of moderate to vigorous activity [13]. Sleep efficiency is the percentage of sleep time out of the total time between bedtime and rise time. The World Health Organization (WHO) recommends that adults should do at least 150 min of moderate-intensity or 75 min of vigorous-intensity activity throughout a week [14].

Patient characteristics, such as age, sex, and FOLFIRINOX chemotherapy specifics, such as start date and number of cycles received, medication use, and follow-up data, were retrieved from medical records by a medical doctor.

### Statistical analysis

EORTC QLQ-C30 questionnaire scores were compared to reference values for cancer patients (*n* = 23,553), stage III–IV cancer patients (*n* = 8,066), liver/bile/pancreas cancer patients (*n* = 750), and general population (*n* = 7,802) with a summary data two sample *t*-test. Reference values were taken from the online dataset of the EORTC Quality of Life Group [15]. The accelerometer raw data files were downloaded and processed with R-package GGIR, version 2.3–0 (http://cran.r-project.org). Repeated measurements of, for example, sleep duration and time spent on activities were averaged per day or night. Correlation between RCSQ questionnaires and EORTC quality of life was tested with Pearson’s correlation coefficient. Questionnaire and Actiwatch results were compared between patients with long (> 12 months) and patients with short (< 12 months) overall survival (OS), calculated from the last day of FOLFIRINOX, with independent samples *t*-tests. Data were analyzed with SPSS Statistics version 25 (IBM, Armonk, NY, USA).

## Results

### Patient characteristics

In total, 41 LAPC patients were included in this study with a median age of 63 years (range 41–76 years). Table 1 presents the patient characteristics. All patients had received at least eight cycles of FOLFIRINOX before inclusion. After FOLFIRINOX, twelve patients (29.3%) showed partial response of the tumor, the other 29 patients (70.7%) stable disease. The median time between the last cycle of FOLFIRINOX and filling out the questionnaires and start of Actiwatch registration was 28 days (range 5–96 days). At the time of analysis, after a median follow-up of 7.9 months, 26 patients (63.4%) had progressive disease, and 14 patients (34.2%) had died.

**Table 1:** Patient characteristics

	LAPC cohort (*n* = 41)
Age (years), median (range)	63 (41–76)
Sex, male (%)	18 (43.9)
WHO performance status (%)	
0	12 (29.3)
1	29 (70.7)
Number of cycles of FOLFIRINOX received (%)	
8	35 (85.4)
9	4 (9.8)
12	2 (4.9)
RECIST^a^ tumor response (%)	
Stable disease	29 (70.7)
Partial response	12 (29.3)
Time between last cycle of FOLFIRINOX and questionnaires (days), median (range)	28 (5–96)
Use of pain medication at time of inclusion, yes (%)	16 (39.0)
Type of pain medication	
Paracetamol	5 (12.2)
Opioids	3 (7.3)
Neuropathic pain medication^b^	1 (2.4)
Paracetamol + NSAIDs	2 (4.9)
Paracetamol + opioids	4 (9.8)
Opioids + neuropathic pain medication	1 (2.4)
EORTC QLQ-C30 questionnaire available (%)	40 (97.6)
Richards-Campbell Sleep Questionnaire available (%)	38 (92.7)
Actiwatch registration (%)	36 (87.8)

*EORTC QLQ-C30* European Organization for Research and Treatment of Cancer Quality of Life Questionnaire, *LAPC* locally advanced pancreatic cancer, *NSAIDs* nonsteroidal anti-inflammatory drugs

^a^According to the RECIST 1.1 criteria for CT scan evaluations

^b^For example, gabapentin or amitriptyline

### Quality of life after FOLFIRINOX treatment

**Fig. 1:**
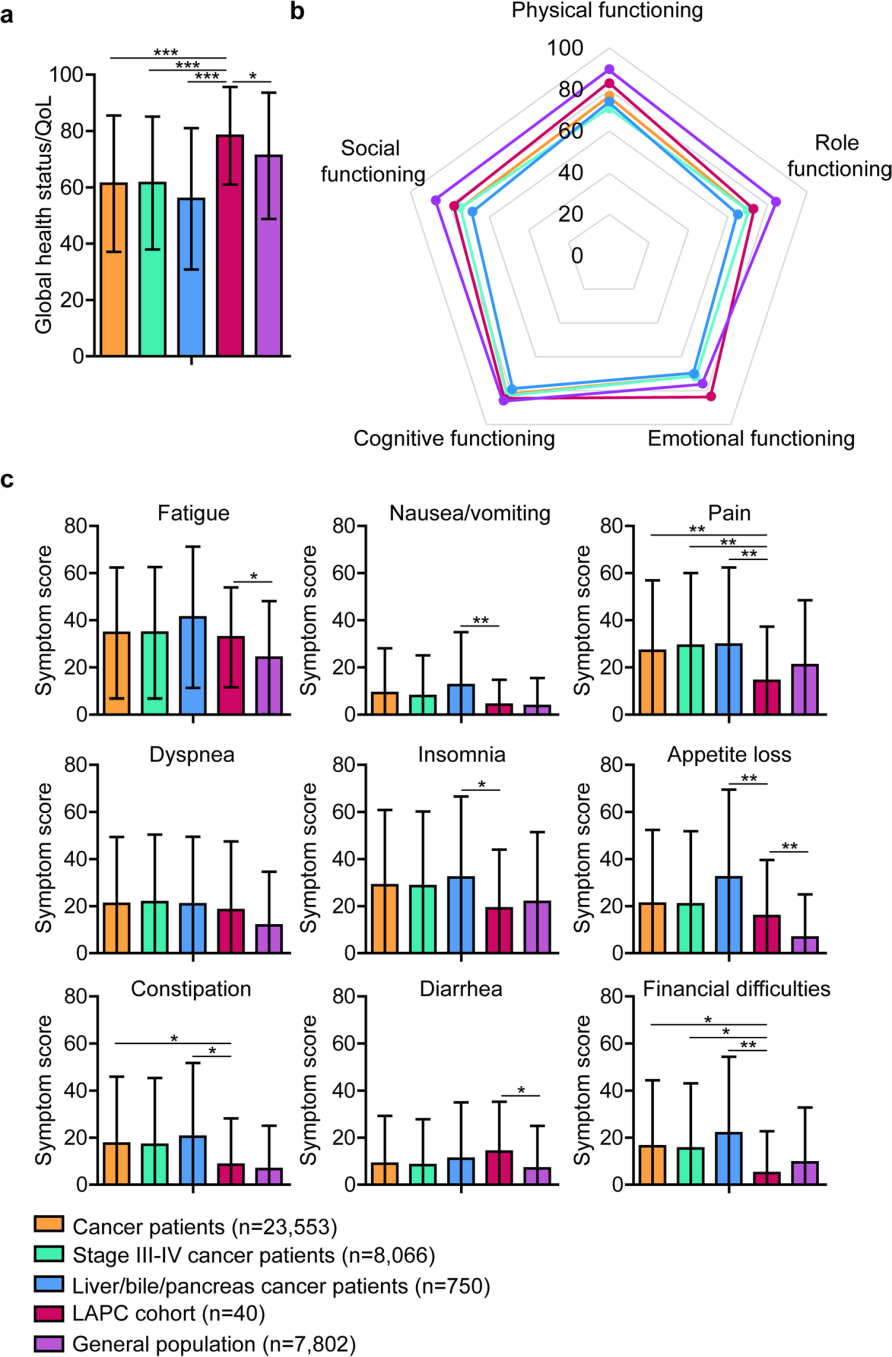
European Organization for Research and Treatment of Cancer (EORTC) Quality of Life Questionnaire (QLQ-C30) outcomes for the locally advanced pancreatic cancer (LAPC) cohort compared to the reference values for cancer patients and general population. **a** Comparison of global health status or quality of life (QoL) between the LAPC cohort and reference cancer patient groups and general population. **b** Scores of functional scales for the LAPC cohort and reference cancer patient groups and general population. **c** Comparison of symptom scores between the LAPC cohort and reference cancer patient groups and general population. Higher scores for global health status and functional scales suggest better quality of life and functioning, and higher scores for symptoms represent more symptoms. *P < 0.05, **P < 0.01, ***P < 0.001

EORTC QLQ-C30 questionnaires were available for 40 patients. One patient withdraw from the study shortly after inclusion and did therefore not fill out any of the questionnaires and did not wear an Actiwatch. The reported answers per questionnaire item can be found in Table 2. The mean score for global health status in this cohort was 78.3 (± standard deviation 17.3). This score was significantly higher than the reported reference values for cancer patients (61.3 ± 24.2, *P* < 0.001), stage III–IV cancer patients (61.5 ± 23.6, *P* < 0.001), liver/bile/pancreas cancer patients (55.9 ± 25.1, *P* < 0.001), and general population (71.2 ± 22.4, *P* = 0.045), as presented in Fig. 1a. In Supplementary Table 3, all EORTC QLQ-C30 scores for our LAPC cohort and reference values are shown.

**Table 2:** Single-item answers to the European Organization for Research and Treatment of Cancer Quality of Life Questionnaire (EORTC QLQ-C30) for the LAPC cohort (*n* = 40)

Item	Category	Not at all, *n* (%)	A little, *n* (%)	Quite a bit, *n* (%)	Very much, *n* (%)
Strenuous activities	PF	12 (30.0)	18 (45.0)	9 (22.5)	0 (0)
Long walk	PF	9 (22.5)	21 (52.5)	8 (20.0)	1 (2.5)
Short walk	PF	30 (75.0)	8 (20.0)	2 (5.0)	0 (0)
Bed or chair	PF	29 (72.5)	11 (27.5)	0 (0)	0 (0)
Self-care	PF	37 (92.5)	3 (7.5)	0 (0)	0 (0)
Limited in work	RF	14 (35.0)	18 (45.0)	6 (15.0)	2 (5.0)
Limited in leisure	RF	21 (52.5)	13 (32.5)	3 (7.5)	3 (7.5)
Dyspnea	DY	26 (65.0)	8 (20.0)	4 (10.0)	2 (5.0)
Pain	PA	24 (60.0)	10 (25.0)	4 (10.0)	2 (5.0)
Need to rest	FA	10 (25.0)	20 (50.0)	9 (22.5)	1 (2.5)
Insomnia	SL	22 (55.0)	14 (35.0)	3 (7.5)	1 (2.5)
Felt weak	FA	12 (30.0)	21 (52.5)	4 (10.0)	3 (7.5)
Appetite loss	AP	25 (62.5)	12 (30.0)	2 (5.0)	1 (2.5)
Nausea	NV	33 (82.5)	7 (17.5)	0 (0)	0 (0)
Vomiting	NV	38 (95.0)	1 (2.5)	1 (2.5)	0 (0)
Constipation	CO	31 (77.5)	7 (17.5)	0 (0)	1 (2.5)
Diarrhea	DI	26 (65.0)	11 (27.5)	3 (7.5)	0 (0)
Felt tired	FA	9 (22.5)	23 (57.5)	8 (20.0)	0 (0)
Pain interference	PA	34 (85..0)	3 (7.5)	2 (5.0)	1 (2.5)
Concentration	CF	25 (62.5)	12 (30.0)	2 (5.0)	0 (0)
Tension	EF	23 (57.5)	15 (37.5)	2 (5.0)	0 (0)
Worry	EF	16 (40.0)	18 (45.0)	6 (15.0)	0 (0)
Irritability	EF	24 (60.0)	15 (37.5)	0 (0)	1 (2.5)
Depression	EF	29 (72.5)	10 (25.0)	1 (2.5)	0 (0)
Memory trouble	CF	23 (57.5)	15 (37.5)	2 (5.0)	0 (0)
Family life	SF	26 (65.0)	10 (25.0)	1 (2.5)	3 (7.5)
Social activities	SF	20 (50.0)	13 (32.5)	3 (7.5)	4 (10.0)
Financial difficulties	FD	36 (90.0)	3 (7.5)	0 (0)	1 (2.5)

*AP* appetite loss, *CF* cognitive functioning, *CO* constipation, *DI* diarrhea, *DY* dyspnea, *EF* emotional functioning, *FA* fatigue, *FD* financial difficulties, *LAPC* locally advanced pancreatic cancer, *NV* nausea and vomiting, *PA* pain, *PF* physical functioning, *RF* role functioning, *SF* social functioning, *SL* insomnia

Patients in this LAPC cohort scored higher on the emotional functioning scale (83.6 ± 16.0) compared to all reference cohorts (*P* < 0.001 for cancer cohorts, *P* = 0.043 for general population), shown in Fig. 1b. LAPC patients also scored higher on the physical functioning scale (83.2 ± 12.4) compared the stage III–IV cancer (*P* = 0.003) and liver/bile/pancreas cancer (*P* = 0.025) reference cohorts. On physical functioning (83.2 ± 12.4 vs 89.8 ± 16.2, *P* = 0.010), role functioning (73.3 ± 27.1 vs 84.7 ± 25.4, *P* = 0.005), and social functioning (78.4 ± 29.2 vs 87.5 ± 22.9, *P* = 0.012), LAPC patients scored lower than the general population reference cohort.

In Fig. 1c reported symptom scores are presented. Compared to the liver/bile/pancreas cancer reference cohort, our LAPC cohort scored lower on the symptom scales for nausea/vomiting (4.2 ± 10.5 vs 14.2 ± 22.5, *P* = 0.005), pain (14.2 ± 23.1 vs 29.6 ± 32.8, *P* = 0.003), insomnia (19.1 ± 24.9 vs 32.2 ± 34.4, *P* = 0.016), appetite loss (15.8 ± 23.8 vs 32.3 ± 37.2, *P* = 0.005), constipation (8.5 ± 19.7 vs 20.4 ± 31.3, *P* = 0.016), and financial difficulties (5.0 ± 17.7 vs 21.9 ± 32.5, *P* = 0.001). Compared to the general population, LAPC patients reported a higher score for fatigue (32.7 ± 21.2 vs 24.1 ± 24.0, *P* = 0.024), appetite loss (15.8 ± 23.8 vs 6.7 ± 18.3, *P* = 0.002), and diarrhea (14.1 ± 21.2 vs 7.0 ± 18.0, *P* = 0.013).

### Pain after FOLFIRINOX treatment

In accordance with the EORTC QLQ-C30 outcome, the LAPC patients in this cohort did not often report symptoms of pain, measured with NRS scores. Only two patients reported an NRS score of > 3: 1 patient NRS 4 and 1 patient NRS 7. The patient with NRS 4 immediately started opioid treatment after inclusion. The patient with NRS 7 showed very early progression of disease, within 2 months after inclusion. Four of the patients reporting any symptoms of pain did not use any pain medication at the time of measurement.

### Sleep quantity and quality after FOLFIRINOX treatment

Objective outcome of sleep, measured with the Actiwatch, was available from 36 patients. Due to technical issues with extraction of the data from the Actiwatch, sleep data was not available for the other four patients. The mean sleep duration was 8.0 h/night (± 1.2 h/night), based on a registration period of seven consecutive nights. The mean sleep efficiency was 69.6% (± 9.0%).

RCSQ questionnaires were available from 38 patients. The questionnaires were filled out during the first five consecutive nights of Actiwatch registration. The mean RCSQ score calculated from all five items during five nights was 72.0 (± 11.4). The scores per item per night are shown in Table 3. Patients reported the lowest scores for sleep depth (mean score 66.2 ± 18.7), and the highest scores for returning to sleep after being awaken (mean score 77.5 ± 11.9).

**Table 3:** Single-item answers to the Richards-Campbell Sleep Questionnaire (RCSQ) for the LAPC cohort (*n* = 38)

Item	Mean score (SD) night 1	Mean score (SD) night 2	Mean score (SD) night 3	Mean score (SD) night 4	Mean score (SD) night 5	Mean score (SD) total period
Sleep depth	64.3 (23.0)	63.1 (26.0)	65.0 (23.6)	69.1 (21.0)	69.2 (22.6)	66.2 (18.7)
Sleep latency	69.1 (23.7)	72.4 (20.2)	75.5 (20.1)	71.1 (24.9)	74.9 (20.6)	72.5 (14.4)
Awakenings	73.3 (20.6)	72.2 (18.3)	73.0 (17.6)	74.2 (17.5)	74.0 (20.7)	73.6 (13.4)
Returning to sleep	77.1 (18.4)	79.3 (13.7)	76.8 (16.9)	77.4 (15.6)	75.2 (20.3)	77.5 (11.9)
Sleep quality	73.8 (21.2)	78.2 (16.3)	75.2 (20.2)	77.5 (17.4)	73.3 (24.1)	75.5 (13.0)
Total score	70.9 (15.3)	71.9 (13.4)	72.5 (16.4)	73.0 (15.9)	72.3 (19.2)	72.0 (11.4)

*LAPC* locally advanced pancreatic cancer, *SD* standard deviation

There was not a significant correlation between patient-reported RCSQ scores and global health status/quality of life (Pearson *r* = 0.18; 95% confidence interval (CI) − 0.17 to 0.48, *P* = 0.306), as presented in Supplementary Fig. 1.

### Activity level after FOLFIRINOX treatment

Objective activity registration, measured with the Actiwatch, was available for 32 patients. For the other eight patients, technical problems with extraction of data made it impossible to analyze activity data. Only 11/32 patients (34.4%) registered a period of moderate to vigorous activity at one or multiple days. The mean duration of moderate or vigorous activity was 5.3 min/day (± 14.8 min/day), based on a registration period of 7 consecutive days. When only including patients with at least one moderate-vigorous activity registered, the mean duration of moderate-vigorous activity was 37 min/week (± 103 min/week). Only three patients (9.4%) did more than 75 min of moderate-vigorous activity during the week, as recommended by the WHO.

### Quality of life in patients with short overall survival after FOLFIRINOX

In Table 4, the most important EORTC QLQ-C30, RCSQ, and Actiwatch results are shown for patients with an overall survival of at least 12 months (*n* = 11) and patients who died within 12 months (*n* = 11) after completion of FOLFIRINOX. There were no differences between groups in patient-reported quality of life, based on the EORTC QLQ-C30 global health status item (mean score 80.4 ± 13.9 for long survival, 76.5 ± 21.5 for short survival patients, *P* = 0.619). There was a difference in patient-reported fatigue (*P* = 0.024): patients with a survival longer than 12 months reported more fatigue symptoms (mean score 45.4 ± 22.7), while patients with a short survival reported lower fatigue symptoms (24.1 ± 17.8). In both groups, 3/11 (27.3%) of patients reported a pain score of NRS > 0. Sleep efficiency, but not sleep duration or sleep quality, was better in patients with OS > 12 months (76.1 ± 5.0%) compared to patients with OS < 12 months (68.3 ± 9.8%, *P* = 0.039).

**Table 4:** Comparison of EORTC QLQ-C30, RCSQ, and Actiwatch results between patients with long and short overall survival (OS) after completion of FOLFIRINOX

	Patients with OS > 12 months (*n* = 11), mean score (SD)	Patients with OS < 12 months (*n* = 11), mean score (SD)	*P*
Global health status (QoL)	80.4 (13.9)	76.5 (21.5)	0.619
Physical functioning	81.2 (15.4)	85.0 (11.1)	0.520
Role functioning	71.2 (30.8)	72.6 (34.4)	0.918
Emotional functioning	82.6 (15.1)	90.8 (13.3)	0.193
Cognitive functioning	80.3 (91.7)	91.7 (14.1)	0.161
Social functioning	63.6 (33.1)	80.5 (26.5)	0.204
Fatigue	45.4 (22.7)	24.1 (17.8)	**0.024**
Nausea and vomiting	4.6 (10.7)	0.0 (0.0)	0.175
Pain	15.3 (23.0)	21.2 (33.4)	0.634
Dyspnea	24.2 (33.7)	21.2 (34.3)	0.838
Insomnia	30.2 (31.5)	9.1 (21.6)	0.082
Appetite loss	24.2 (33.7)	9.0 (15.4)	0.189
Constipation	12.1 (30.8)	6.0 (13.4)	0.554
Diarrhea	15.1 (22.9)	12.0 (16.7)	0.721
Financial difficulties	12.1 (30.8)	6.0 (13.4)	0.554
Pain score NRS > 0, yes (%)	3 (27.3)	3 (27.3)	1.000
Sleep duration, hours	8.6 (0.9)	7.8 (1.4)	0.142
Sleep efficiency (%)	76.1 (5.0)	68.3 (9.8)	**0.039**
Sleep quality (RCSQ)	72.4 (15.2)	74.0 (10.7)	0.783

*P*-values in bold are statistically significant

*EORTC QLQ-C30* European Organization for Research and Treatment of Cancer Quality of Life Questionnaire, *QoL* quality of life, *RCSQ* Richards-Campbell Sleep Questionnaire, *SD* standard deviation

## Discussion

In this representative cohort with a median age of 63 years, we investigated the quality of life of LAPC patients after completion of FOLFIRINOX chemotherapy. We found that patients within our LAPC cohort reported high quality of life scores and low symptom scores, measured with the EORTC QLQ-C30 quality of life questionnaire. Quality of life scores were better than the reported scores for cancer reference cohorts and even better than general population references. LAPC patients reported more symptoms of fatigue, appetite loss, and diarrhea compared to the general population, but less frequent than cancer reference cohorts, including PDAC patients. The majority of LAPC patients (73%) reported no pain symptoms, with or without the use of pain medication. Patients also showed sufficient sleep duration (8 h/night) and sleep efficiency (70%), objectively measured by Actiwatch registration, and they reported high quality of sleep. The activity level of LAPC patients was, however, very low. A minority of patients in this cohort did some moderate to vigorous activity during the registration period. Only three patients reached the activity level recommended by the WHO. The activity level of this LAPC cohort is lower compared to previous published data on activity levels in patients with different types of cancer (e.g., lymphoma, breast cancer, head and neck cancer, colon cancer) [16].

Not many studies have published on quality of life in PDAC patients during or after treatment. A systematic review on the incidence and overall burden of PDAC in Europe showed, based on data from three different cohorts, that PDAC patients report a worse quality of life compared to the general population [17]. This data, however, was retrieved at time of diagnosis from patients of all disease stages, before start of any treatment, and is, therefore, not comparable to our data. Studies that investigated the change in quality of life after treatment, measured with the EORTC QLQ-C30, all showed better results after treatment compared to baseline measurements. Patients with resectable disease showed improving quality of life results after operation, despite extensive surgical procedures [18, 19]. Also in metastatic disease patients, included in a phase II trial to investigate the response to and toxicity of FOLFIRINOX, quality of life improved after treatment [20]. One study showed that patients eligible for (chemotherapy) treatment report better quality of life compared to those who only received best supportive care [21]. Also, patients treated with palliative care were more satisfied with the given care than patients treated with curative intent [18].

Our results are in line with the literature. We hypothesize that PDAC patients in this cohort, despite their poor prognosis, reported good to excellent quality of life for a couple of reasons. First, patients in this study all finished treatment and toxicity-related symptoms of FOLFIRINOX seem to pass by quickly after treatment is completed. Unfortunately, we do not have data on quality of life during treatment, but it is to be assumed that quality of life is better after FOLFIRINOX than during this toxic chemotherapy. Second, in patient responding to treatment, FOLFIRINOX will diminish the disease load and by that the symptom burden [22–24]. This is affirmed by the results of other quality of life studies in PDAC patients after treatment [18–21]. Third, undergoing full treatment might also have psychological benefit. Any treatment, standard chemotherapy, or experimental immunotherapy for example, will give hope for curation or improved survival. Instead of waiting for disease progression, these patients have done everything they can to improve their life expectancy. Also, previous research has shown that patients participating in clinical trials receive better quality of care resulting in better patient outcome [25, 26]. Finally, these patients answered the questionnaires several months after diagnosis. By that time, they might have come to peace with the disease and its prognosis. Because of these positive results, we believe that treating physicians should maybe be less reluctant in considering FOLFIRINOX as treatment in LAPC patients.

A limitation of this study is that we could not include all patient who have started FOLFIRINOX. However, in our previous cohort study, only 12% of patients with LAPC showed progressive disease during FOLFIRINOX treatment, the vast majority had stable disease [27], and the effectiveness of FOLFIRINOX as first-line treatment for LAPC patients has been extensively published [1, 3]. Also, out of 41 patients included in this clinical trial, ten received first-line FOLFIRINOX within the Erasmus Medical Center Rotterdam between October 2019 and August 2020. During that time, a total of fifteen patients started FOLFIRINOX in this hospital of which only three (20%) showed progression of disease and could therefore not participate in the trial. Eligibility data of patients from other centers was not available, but we believe those will be similar to the EMC data. Based on these results, we believe that the cohort described in our current study is not a rare patient population tolerating FOLFIRINOX very well with measurable tumor response on CT scans but represent the vast majority of LAPC patients treated with this chemotherapy regimen.

This is the first study on quality of life in LAPC patients treated with FOLFIRINOX, and the results can be used to help inform patients during shared decision-making. However, our data is based on a relatively small sample size (*n* = 41) with no comparison to baseline data or data at time of diagnosis. Also, patient outcome might be positively biased because all patients included in this clinical trial showed at least stable disease after FOLFIRINOX treatment. It would be interesting to investigate the quality of life of patients with progressive disease during treatment in the future.

## Conclusion

LAPC patients with disease control after FOLFIRINOX treatment report very good quality of life. They report very little symptoms or pain and good sleep quality and sleep duration after treatment with FOLFIRINOX.

## Supplementary Information

Below is the link to the electronic supplementary material.

Supplementary file1 (DOCX 93 KB)

## Data Availability

Data are available from the authors upon reasonable request at the corresponding author and with permission of the Erasmus Medical Center Rotterdam.

## References

[1] Suker M, Beumer BR, Sadot E, Marthey L, Faris JE, Mellon EA (2016). FOLFIRINOX for locally advanced pancreatic cancer: a systematic review and patient-level meta-analysis. Lancet Oncol.

[2] Chan K, Shah K, Lien K, Coyle D, Lam H, Ko YJ (2014). A Bayesian meta-analysis of multiple treatment comparisons of systemic regimens for advanced pancreatic cancer. PLoS One.

[3] Rombouts SJ, Walma MS, Vogel JA, van Rijssen LB, Wilmink JW, Mohammad NH (2016). Systematic review of resection rates and clinical outcomes after FOLFIRINOX-based treatment in patients with locally advanced pancreatic cancer. Ann Surg Oncol.

[4] Rombouts SJ, Mungroop TH, Heilmann MN, van Laarhoven HW, Busch OR, Molenaar IQ (2016). FOLFIRINOX in locally advanced and metastatic pancreatic cancer: a single centre cohort study. J Cancer.

[5] Fest J, Ruiter R, van Rooij FJ, van der Geest LG, Lemmens VE, Ikram MA (2017). Underestimation of pancreatic cancer in the national cancer registry - reconsidering the incidence and survival rates. Eur J Cancer.

[6] Thibodeau S, Voutsadakis IA (2018) FOLFIRINOX chemotherapy in metastatic pancreatic cancer: a systematic review and meta-analysis of retrospective and phase II studies. J Clin Med 2018 Jan 4;7(1):7 10.3390/jcm7010007 PMC5791015 29300345

[7] Drewes AM, Campbell CM, Ceyhan GO, Delhaye M, Garg PK, van Goor H (2018). Pain in pancreatic ductal adenocarcinoma: a multidisciplinary, International guideline for optimized management. Pancreatology.

[8] Boyd AD, Brown D, Henrickson C, Hampton J, Zhu B, Almani F (2012). Screening for depression, sleep-related disturbances, and anxiety in patients with adenocarcinoma of the pancreas: a preliminary study. ScientificWorldJournal.

[9] Aaronson NK, Ahmedzai S, Bergman B, Bullinger M, Cull A, Duez NJ (1993). The European Organization for Research and Treatment of Cancer QLQ-C30: a quality-of-life instrument for use in international clinical trials in oncology. J Natl Cancer Inst.

[10] Jeffs EL, Darbyshire JL (2019). Measuring sleep in the intensive care unit: a critical appraisal of the use of subjective methods. J Intensive Care Med.

[11] Richards KC, O'Sullivan PS, Phillips RL (2000). Measurement of sleep in critically ill patients. J Nurs Meas.

[12] Karaman Özlü Z, Şahin Altun Ö, Olçun Z, Kaya M, Yurttaş A (2018). Examination of the relationship between elective surgical patients' methods for coping with stress and sleeping status the night before an operation. J Perianesth Nurs.

[13] Pavey TG, Gomersall SR, Clark BK, Brown WJ (2016). The validity of the GENEActiv wrist-worn accelerometer for measuring adult sedentary time in free living. J Sci Med Sport.

[14] Bull FC, Al-Ansari SS, Biddle S, Borodulin K, Buman MP, Cardon G (2020). World Health Organization 2020 guidelines on physical activity and sedentary behaviour. Br J Sports Med.

[15] Scott NW, Fayers PM, Aaronson NK, Bottomley A, De Graeff A, Groenvold M, et al (2008) EORTC QLQ-C30 Reference Values Manual (2nd ed.) EORTC Quality of Life Group. http://groups.eortc.be/qol/downloads/reference_values_manual2008.pdf

[16] Douma JAJ, de Beaufort MB, Kampshoff CS, Persoon S, Vermaire JA, Chinapaw MJ (2020). Physical activity in patients with cancer: self-report versus accelerometer assessments. Support Care Cancer.

[17] Carrato A, Falcone A, Ducreux M, Valle JW, Parnaby A, Djazouli K (2015). A systematic review of the burden of pancreatic cancer in europe: real-world impact on survival, quality of life and costs. J Gastrointest Cancer.

[18] Mackay TM, van Rijssen LB, Andriessen JO, Suker M, Creemers GJ, Eskens FA (2020). Patient Satisfaction and quality of life before and after treatment of pancreatic and periampullary cancer: a prospective multicenter study. J Natl Compr Canc Netw.

[19] Crippa S, Domínguez I, Rodríguez JR, Razo O, Thayer SP, Ryan DP (2008). Quality of life in pancreatic cancer: analysis by stage and treatment. J Gastrointest Surg.

[20] Conroy T, Paillot B, François E, Bugat R, Jacob JH, Stein U (2005). Irinotecan plus oxaliplatin and leucovorin-modulated fluorouracil in advanced pancreatic cancer–a Groupe Tumeurs Digestives of the Federation Nationale des Centres de Lutte Contre le Cancer study. J Clin Oncol.

[21] Glimelius B, Hoffman K, Sjödén PO, Jacobsson G, Sellström H, Enander LK (1996). Chemotherapy improves survival and quality of life in advanced pancreatic and biliary cancer. Ann Oncol.

[22] Shi Q, Smith TG, Michonski JD, Stein KD, Kaw C, Cleeland CS (2011). Symptom burden in cancer survivors 1 year after diagnosis: a report from the American Cancer Society's Studies of Cancer Survivors. Cancer.

[23] van Kleef JJ, Ter Veer E, van den Boorn HG, Schokker S, Ngai LL, Prins MJ (2020). Quality of life during palliative systemic therapy for esophagogastric cancer: systematic review and meta-analysis. J Natl Cancer Inst.

[24] Thong MS, Mols F, Lemmens VE, Creemers GJ, Slooter GD, van de Poll-Franse LV (2011). Impact of chemotherapy on health status and symptom burden of colon cancer survivors: a population-based study. Eur J Cancer.

[25] Janni W, Kiechle M, Sommer H, Rack B, Gauger K, Heinrigs M (2006). Study participation improves treatment strategies and individual patient care in participating centers. Anticancer Res.

[26] Majumdar SR, Roe MT, Peterson ED, Chen AY, Gibler WB, Armstrong PW (2008). Better outcomes for patients treated at hospitals that participate in clinical trials. Arch Intern Med.

[27] Suker M, Nuyttens JJ, Eskens F, Haberkorn BCM, Coene PLO, van der Harst E (2019). Efficacy and feasibility of stereotactic radiotherapy after folfirinox in patients with locally advanced pancreatic cancer (LAPC-1 trial). EClinicalMedicine.

