# Supplementary material for: Quality of life of locally advanced pancreatic cancer patients after FOLFIRINOX treatment
Source: Support Care Cancer. 2021 Nov 11;30(3):2407–15. doi: 10.1007/s00520-021-06648-1 (PMC8794891; doi:10.1007/s00520-021-06648-1)
Supplement: Supplementary file 1 — Supplementary file1 (DOCX 93 KB) [file 520_2021_6648_MOESM1_ESM.docx]

**Supplementary Table 1.** European Organization for Research and Treatment of Cancer (EORTC) Quality of Life Questionnaire (QLQ-C30), English language version 3.

| **Item** | **Not at all** | | **A little** | | | **Quite a bit** | | | **Very much** | |
| --- | --- | --- | --- | --- | --- | --- | --- | --- | --- | --- |
| 1. Do you have any trouble doing strenuous activities, like carrying a heavy shopping bag or a suitcase? | **1** | | **2** | | | **3** | | | **4** | |
| 2. Do you have any trouble taking a long walk? | **1** | | **2** | | | **3** | | | **4** | |
| 3. Do you have any trouble taking a short walk outside of the house? | **1** | | **2** | | | **3** | | | **4** | |
| 4. Do you need to stay in bed or a chair during the day? | **1** | | **2** | | | **3** | | | **4** | |
| 5. Do you need help with eating, dressing, washing yourself or using the toilet? | **1** | | **2** | | | **3** | | | **4** | |
| **During the past week:** | | | | | | | | | | |
| 6. Were you limited in doing either your work or other daily activities? | **1** | | **2** | | | **3** | | | **4** | |
| 7. Were you limited in pursuing your hobbies or other leisure time activities? | **1** | | **2** | | | **3** | | | **4** | |
| 8. Were you short of breath? | **1** | | **2** | | | **3** | | | **4** | |
| 9. Have you had pain? | **1** | | **2** | | | **3** | | | **4** | |
| 10. Did you need to rest? | **1** | | **2** | | | **3** | | | **4** | |
| 11. Have you had trouble sleeping? | **1** | | **2** | | | **3** | | | **4** | |
| 12. Have you felt weak? | **1** | | **2** | | | **3** | | | **4** | |
| 13. Have you lacked appetite? | **1** | | **2** | | | **3** | | | **4** | |
| 14. Have you felt nauseated? | **1** | | **2** | | | **3** | | | **4** | |
| 15. Have you vomited? | **1** | | **2** | | | **3** | | | **4** | |
| 16. Have you been constipated? | **1** | | **2** | | | **3** | | | **4** | |
| 17. Have you had diarrhea? | **1** | | **2** | | | **3** | | | **4** | |
| 18. Were you tired? | **1** | | **2** | | | **3** | | | **4** | |
| 19. Did pain interfere with your daily activities? | **1** | | **2** | | | **3** | | | **4** | |
| 20. Have you had difficulty in concentrating on things, like reading a newspaper or watching television? | **1** | | **2** | | | **3** | | | **4** | |
| 21. Did you feel tense? | **1** | | **2** | | | **3** | | | **4** | |
| 22. Did you worry? | **1** | | **2** | | | **3** | | | **4** | |
| 23. Did you feel irritable? | **1** | | **2** | | | **3** | | | **4** | |
| 24. Did you feel depressed? | **1** | | **2** | | | **3** | | | **4** | |
| 25. Have you had difficulty remembering things? | **1** | | **2** | | | **3** | | | **4** | |
| 26. Has your physical condition or medical treatment interfered with your family life? | **1** | | **2** | | | **3** | | | **4** | |
| 27. Has your physical condition or medical treatment interfered with your social activities? | **1** | | **2** | | | **3** | | | **4** | |
| 28. Has your physical condition or medical treatment caused you financial difficulties? | **1** | | **2** | | | **3** | | | **4** | |
| **For the following questions please circle the number between 1 and 7 that best applies to you** | | | | | | | | | | |
| 29. How would you rate your overall health during the past week? | **1** | **2** | | **3** | **4** | | **5** | **6** | | **7** |
| 30. How would you rate your overall quality of life during the past week? | **1** | **2** | | **3** | **4** | | **5** | **6** | | **7** |

**Supplementary Table 2.** Richard Campbell Sleep Questionnaire (RCSQ), English language version.

| **Item** | **Question** | **Answer*** | |
| --- | --- | --- | --- |
| **Sleep depth** | My sleep last night was: | Light sleep (0) | Deep sleep (100) |
| **Sleep latency** | Last night, the first time I got to sleep, I: | Just never could fall asleep (0) | Fell asleep almost immediately (100) |
| **Awakenings** | Last night, I was: | Awake all night long (0) | Awake very little (100) |
| **Returning to sleep** | Last night, when I woke up or was awakened, I: | Couldn’t get back to sleep (0) | Got back to sleep immediately (100) |
| **Sleep quality** | I would describe my sleep last night as: | A bad night’s sleep (0) | A good night’s sleep (100) |

* Each question is scored on a 100-mm visual analogue scale.

**Supplementary Table 3.** Scores of the European Organization for Research and Treatment of Cancer (EORTC) Quality of Life Questionnaire (QLQ-C30) for the LAPC cohort compared to the reference values for cancer patients and general population.

|  | **LAPC cohort (n=40), mean score (SD)** | **Cancer patients (n=23,553), mean score (SD)** | ***P*** | **Stage III-IV cancer patients (n=8,066), mean score (SD)** | ***P*** | **Liver/bile/**  **pancreas cancer patients (n=750), mean score (SD)** | ***P*** | **General population (n=7,802), mean score (SD)** | ***P*** |
| --- | --- | --- | --- | --- | --- | --- | --- | --- | --- |
| **Global health status (QoL)** | 78.3 (17.3) | 61.3 (24.2) | **<0.001** | 61.5 (23.6) | **<0.001** | 55.9 (25.1) | **<0.001** | 71.2 (22.4) | **0.045** |
|  | |  | | | | | | | |
| **Physical functioning** | 83.2 (12.4) | 76.7 (23.2) | 0.076 | 71.2 (25.8) | **0.003** | 74.1 (25.7) | **0.025** | 89.8 (16.2) | **0.010** |
| **Role functioning** | 73.3 (27.1) | 70.5 (32.8) | 0.589 | 70.6 (32.8) | 0.603 | 65.2 (36.3) | 0.159 | 84.7 (25.4) | **0.005** |
| **Emotional functioning** | 83.6 (16.0) | 71.4 (24.2) | **0.001** | 71.5 (23.8) | **0.001** | 69.8 (25.5) | **0.001** | 76.3 (22.8) | **0.043** |
| **Cognitive functioning** | 85.0 (17.0) | 82.6 (21.9) | 0.488 | 83.2 (21.3) | 0.594 | 79.0 (23.1) | 0.101 | 86.1 (20.0) | 0.728 |
| **Social functioning** | 78.4 (29.2) | 75.0 (29.1) | 0.460 | 75.1 (28.9) | 0.471 | 69.0 (31.7) | 0.061 | 87.5 (22.9) | **0.012** |
|  | |  | | | | | | | |
| **Fatigue** | 32.7 (21.2) | 34.6 (27.8) | 0.666 | 34.7 (27.9) | 0.651 | 41.2 (30.0) | 0.074 | 24.1 (24.0) | **0.024** |
| **Nausea and vomiting** | 4.2 (10.5) | 9.1 (19.0) | 0.103 | 7.8 (17.3) | 0.189 | 14.2 (22.5) | **0.005** | 3.7 (11.7) | 0.787 |
| **Pain** | 14.2 (23.1) | 27.0 (29.9) | **0.007** | 29.2 (30.8) | **0.002** | 29.6 (32.8) | **0.003** | 20.9 (27.6) | 0.125 |
| **Dyspnea** | 18.3 (29.2) | 21.0 (28.4) | 0.548 | 21.7 (28.7) | 0.455 | 20.8 (28.7) | 0.583 | 11.8 (22.8) | 0.073 |
| **Insomnia** | 19.1 (24.9) | 28.9 (31.9) | 0.052 | 28.5 (31.7) | 0.061 | 32.2 (34.4) | **0.016** | 21.8 (29.7) | 0.566 |
| **Appetite loss** | 15.8 (23.8) | 21.1 (31.3) | 0.284 | 20.8 (31.0) | 0.308 | 32.3 (37.2) | **0.005** | 6.7 (18.3) | **0.002** |
| **Constipation** | 8.5 (19.7) | 17.5 (28.4) | **0.045** | 17.0 (28.4) | 0.059 | 20.4 (31.3) | **0.016** | 6.7 (18.4) | 0.537 |
| **Diarrhea** | 14.1 (21.2) | 9.0 (20.3) | 0.112 | 8.3 (19.5) | 0.061 | 11.1 (23.9) | 0.428 | 7.0 (18.0) | **0.013** |
| **Financial difficulties** | 5.0 (17.7) | 16.3 (28.1) | **0.011** | 15.4 (27.7) | **0.018** | 21.9 (32.5) | **0.001** | 9.5 (23.3) | 0.223 |

LAPC = locally advanced pancreatic cancer, QoL = quality of life, SD = standard deviation.


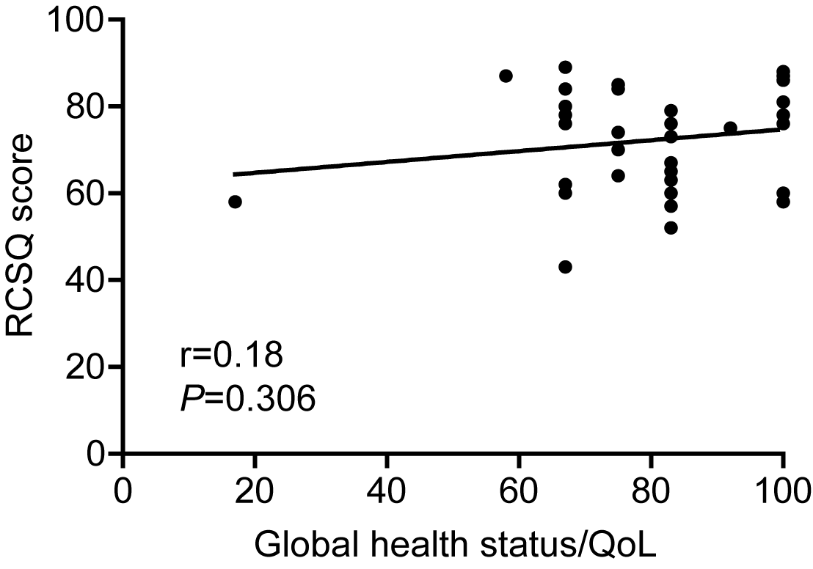


**Supplementary Figure 1.** Correlation plot of Richard Campbell Sleep Questionnaire (RCSQ) scores and global health score/quality of life as measured with the European Organization for Research and Treatment of Cancer (EORTC) Quality of Life Questionnaire (QLQ-C30) questionnaire. There was no significant correlation (*P*=0.306) between patient-reported sleep quality and patient-reported quality of life (Pearson’s r=0.18; 95% confidence interval (CI) -0.17-0.48).
